# Phase-Coherence Transitions and Communication in the Gamma Range between Delay-Coupled Neuronal Populations

**DOI:** 10.1371/journal.pcbi.1003723

**Published:** 2014-07-24

**Authors:** Alessandro Barardi, Belen Sancristóbal, Jordi Garcia-Ojalvo

**Affiliations:** 1 Departament of Experimental and Health Sciences, Universitat Pompeu Fabra, Barcelona Biomedical Research Park, Barcelona, Spain; 2 Departament de Física i Enginyeria Nuclear, Universitat Politècnica de Catalunya, Terrassa, Spain; 3 Center for Genomic Regulation, Barcelona Biomedical Research Park, Barcelona, Spain; University of Toronto, Canada

## Abstract

Synchronization between neuronal populations plays an important role in information transmission between brain areas. In particular, collective oscillations emerging from the synchronized activity of thousands of neurons can increase the functional connectivity between neural assemblies by coherently coordinating their phases. This synchrony of neuronal activity can take place within a cortical patch or between different cortical regions. While short-range interactions between neurons involve just a few milliseconds, communication through long-range projections between different regions could take up to tens of milliseconds. How these heterogeneous transmission delays affect communication between neuronal populations is not well known. To address this question, we have studied the dynamics of two bidirectionally delayed-coupled neuronal populations using conductance-based spiking models, examining how different synaptic delays give rise to in-phase/anti-phase transitions at particular frequencies within the gamma range, and how this behavior is related to the phase coherence between the two populations at different frequencies. We have used spectral analysis and information theory to quantify the information exchanged between the two networks. For different transmission delays between the two coupled populations, we analyze how the local field potential and multi-unit activity calculated from one population convey information in response to a set of external inputs applied to the other population. The results confirm that zero-lag synchronization maximizes information transmission, although out-of-phase synchronization allows for efficient communication provided the coupling delay, the phase lag between the populations, and the frequency of the oscillations are properly matched.

## Author Summary

The correct operation of the brain requires a carefully orchestrated activity, which includes the establishment of synchronized behavior among multiple neuronal populations. Synchronization of collective neuronal oscillations, in particular, has been suggested to mediate communication between brain areas, with the global oscillations acting as “information carriers” on which signals encoding specific stimuli or brain states are superimposed. But neuronal signals travel at finite speeds across the brain, thus leading to a wide range of delays in the coupling between neuronal populations. How the brain reaches the required level of coordination in the presence of such delays is still unclear. Here we approach this question in the case of two delay-coupled neuronal populations exhibiting collective oscillations in the gamma range. Our results show that effective communication can be reached even in the presence of relatively large delays between the populations, which self-organize in either in-phase or anti-phase synchronized states. In those states the transmission delays, phase difference, and oscillation frequency match to allow for communication at a wide range of coupling delays between brain areas.

## Introduction

Brain function emerges from the collective dynamics of coupled neurons, the structural connectivity among which enables correlations between their firing activities. As a result of these correlations, effective neuronal networks function collectively on a mesoscopic scale, comprising thousands of coupled neurons that operate together, giving rise to emergent behavior. In awake animals, this collective dynamics takes the form of recurrent series of high and low network activity, corresponding with repetitive epochs of increased excitation over inhibition followed by boosts of inhibition. This leads to the appearance of rhythmicity at certain frequency bands. In particular, oscillations in the gamma-band (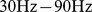) are observed in several cortical areas in relation with cognitive tasks [1].

Synchronized oscillations can increase the functional connectivity between neural assemblies by coherently coordinating their firing dynamics. This hypothesis, known as communication through coherence (CTC), was proposed [2] as a mechanism by which gamma-band synchronization could regulate routing of information between brain areas. Since neuronal oscillations are associated with the dynamics of the excitatory-inhibitory balance, they represent periodic modulations of the excitability of neurons, which are more likely to spike within specific time windows (i.e. when inhibition is low). If two neuronal populations oscillate with a constant phase difference, then an effective transmission of information between the two groups of neurons is achieved provided the spikes sent by a population reach systematically the other population at the peaks of excitability. In that way, modulation of the relative phases of the emerging rhythms might dynamically generate functional cell assemblies [3]–[5].

A key requirement of the CTC mechanism is the existence of a constant phase difference between the two neuronal networks that reliably allows their binding, favoring communication. This coordination can be expected to arise from the synaptic coupling between the neurons of the two populations. But this coupling is not instantaneous, since propagation times between different cortical regions can reach up to several tens of milliseconds [6]. Previous CTC studies have mainly concentrated on describing the dependence of the coherence on the phase lag between the neuronal populations [2], [3], [7], without examining systematically the relationship between the actual coupling delay and the phase lag at which the coherence is maximal. In fact, coupled nonlinear oscillators are known to adjust their phases upon frequency locking, leading under certain conditions to either in-phase (zero phase lag) or anti-phase synchronization (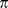-phase lag) [8]. Anti-phase patterns in cortical networks, for instance, have been widely studied [9]. Zero-lag synchronization, in turn, has been experimentally observed between gamma oscillations emerging from separated brain areas [10]–[12]. The conditions leading to zero-lag synchronization in neuronal oscillations are however somewhat stringent, requiring non-trivial spiking dynamics [13] or complex network architectures [14], [15]. In particular, zero-lag synchronization between two cortical areas has been shown to be possible even with long axonal delays [15], [16], provided the two areas interact through a third oscillator, which could correspond to the thalamus [17], [18].

But in contrast with most nonlinear oscillators neuronal populations are highly complex, since they contain a very large number of degrees of freedom (corresponding to the individual neurons), their oscillations are a pure collective phenomenon (the individual neurons in the population do not oscillate), and they operate in a broad frequency range. Additionally, neuronal populations are connected by a large number of axons, and inhomogeneities in the properties of those axons affect differentially the propagation speed of action potentials and lead to a wide spectrum of axonal delays rather than a uniform distribution [19]. It thus becomes necessary to study systematically the conditions under which two such complex oscillators synchronize (i.e. lock their frequencies), what is the resulting phase difference between them, how does this phase difference relate with the coupling delay (and with the frequency band being considered), and how is the efficiency of the communication between the two cortical areas affected by the delayed coupling. We address these questions in what follows.

As mentioned above, within the CTC scenario effective communication arises when spikes from the emitting neuronal population reach the receiver population during the windows of maximum excitability. For this to happen two conditions have to be met: (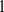) the two coupled oscillators should be frequency locked, and (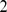) the transmission delay, the oscillation frequency, and the phase difference between the two oscillations should match. In particular, if the networks and the inter-connectivity is symmetric the second condition should hold in the two directions of spike propagation. The time delay (or rather, the distribution of time delays) is fixed as given by the anatomical connectivity. Therefore, it is the frequency of the oscillation spectrum what determines the particular phase lag that meets the matching condition. We have investigated whether this condition only occurs at specific rhythms, or if it holds at all frequencies. To this aim, we have represented mathematically two reciprocally connected identical neuronal populations using conductance-based models for both excitatory and inhibitory cells, and have studied how the heterogeneous axonal delays between the populations affect their synchronization.

We have characterized the collective dynamics through a variable comparable to the local field potential (LFP) recordings [20]. In agreement with experimental data, the power of the modeled LFP decays with increasing frequencies [21]. Here we have focused on the particular dynamical regime in which the collective oscillations show a prominent contribution in the gamma range arising from the inhibitory (GABAergic) synaptic decay time constants [22]. Lower frequency bands contain a strong component arising from the noisy Poissonian distribution of interspike intervals (ISI), which affect the synaptic activation and hence do not reflect the intrinsic dynamics of the network. On the contrary, higher frequency bands of small amplitude reflect the fast dynamics of the action potentials, also affecting the synapse activation time course.

**Figure 1:**
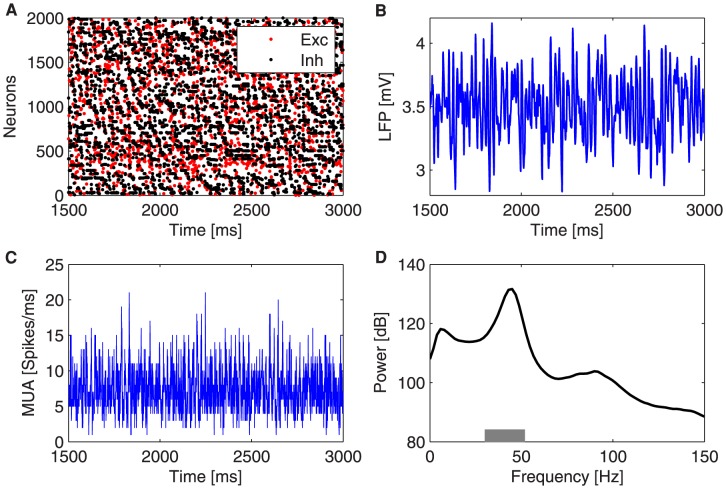
Collective oscillations of a population of 2000 neurons. (A) Raster plot of 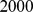 neurons (in red the excitatory and in black the inhibitory neurons) for a 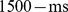 interval. (B) LFP time trace in a 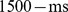 interval for an external mean rate of 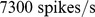. (C) MUA signal calculated counting the number of spikes of the neural population per unit time. (D) LFP power spectrum calculated using the Welch method averaged over 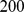 trials. The gray horizontal bar delimits the gamma peak band (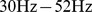).

The modeled neuronal networks exhibit other well-known features of cortical dynamics, such as coexistence of irregular firing at the single-neuron level with collective rhythmicity at the population level, arising from the synaptic recurrent connections between the excitatory and inhibitory neurons [23] (see Figure 1). The excitatory and inhibitory synaptic currents are balanced by compensating the higher number of excitatory neurons (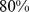 of the whole network) with fast spiking inhibitory neurons and with strong inhibitory synaptic conductances. As a consequence, the neurons remain excitable but spent most of their time with a membrane voltage that fluctuates under the firing threshold. The gamma rhythm emerges from the periodic changes of this balanced synaptic current, which leads to periodic modulation of the distance to threshold. We have characterized the global activity of the network by means of averaging measures such as the aforementioned local field potential (LFP) and the multi-unit activity (MUA). We first used these measures to quantify phase coherence between the oscillatory activity of the two delay-coupled populations at varying mean axonal delays, observing transitions between in-phase and anti-phase dynamics. We next used information theory to quantify the response of one population (the receiver) to a varying external input impinging originally on the other population (the emitter). Our results show that information transmission is enhanced at zero-lag (in-phase) synchronization, and decreases at long delays for which communication occurs through anti-phase dynamics.

## Results

### In-phase synchronization of collective oscillations under instantaneous coupling

We start by considering an isolated population of 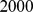 neurons, of which 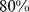 are excitatory and 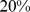 are inhibitory. Each neuron forms on average 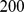 random connections within the network, and all pairs of coupled neurons exhibit a certain time delay, taken from a gamma distribution whose scale and shape parameters are both equal to unity. All neurons receive an external Poisson-distributed spike train whose instantaneous firing rate follows an Ornstein-Uhlenbeck process with a mean value set to 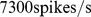. This input and the excitatory recurrent synaptic activity are balanced by the recurrent inhibitory synaptic flow, since the GABAergic conductances are stronger than the glutamatergic AMPA ones. Furthermore, the inhibitory neurons fire at higher rates than the excitatory cells. Therefore, the membrane voltage of the neurons fluctuates below threshold, occasionally crossing it [24]. Despite the fact that the neurons fire sparsely and irregularly (see Figure 1A), a rhythmicity emerges when considering the dynamics of multiple action potentials elicited by thousands of neurons [23]. These oscillations represent the transient synchronized activity of neuronal assemblies, and can be revealed by population measures such as the local field potential (Figure 1B) and the multi-unit activity (Figure 1C), defined in the Materials and Methods section. In the computational model used throughout this work, the collective oscillatory dynamics exhibit a prominent gamma rhythm (Figure 1D), whose period is mainly determined by the decay time constant of inhibition [23], [25], [26].

**Figure 2:**
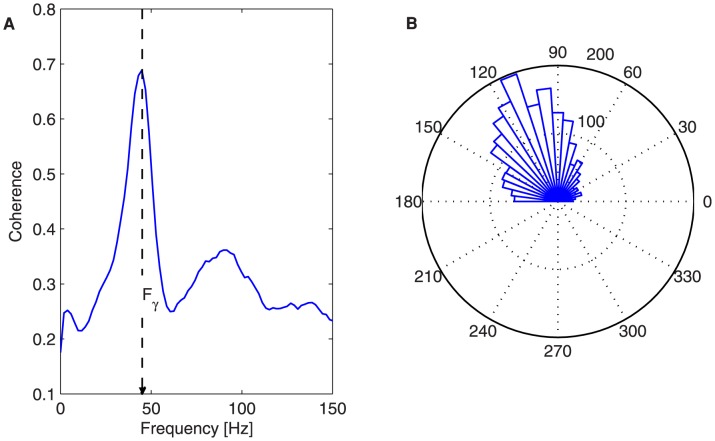
Phase locking between LFP and MUA of a network. (A) LFP-MUA phase coherence for a single population. (B) Angle histogram of the phase difference between the LFP and MUA. The measures are averaged over 200 trials.

Another way of understanding the emergent gamma oscillations is by looking at the coupling between the MUA and the LFP. Since the LFP mainly captures the synaptic currents impinging on the pyramidal neurons (see Materials and Methods section), it is a measure of the excitability of the network. Hence, at those intervals in which inhibition is low (i.e. the inhibitory synaptic current fades away), the probability of firing is high. Due to the recurrent connections between the excitatory and inhibitory neurons, both the initiation and termination of the population bursts occur with a certain periodicity. Here this oscillatory pattern is around 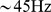 due to the inhibitory decay time constants [22]. The LFP and MUA are mutually locked to this frequency (Figure 2A), and the spikes occur with higher probability close to the troughs of the LFP (i.e. the minimum of inhibition, Figure 2B).

We next consider two bidirectionally coupled neuronal networks of the type described above. Connections between the two areas are excitatory: 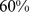 of the excitatory neurons of each network project randomly to 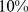 of the neurons belonging to the other pool. Although these parameter values cannot be generalized to any two separate brain areas, for which the specific connectivity might determine their interactions, it is known that the probability of connection decays with distance [27]–[29]. Here we assume that the connectivity within a network is 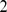-fold the connectivity across networks, neglecting heterogeneity across neurons. Moreover, in order to obtain a certain amount of phase coherence between the two networks, we consider that the majority of excitatory neurons project onto the other network. A stronger (weaker) coupling will lead to unrealistically higher (lower) phase coherence values [30]. We have introduced time delays in the coupling between networks, assuming that the inter-areal delays are larger than the intra-areal delays due to long-range connections. We also consider that the inter-areal delays are distributed heterogenously across the system [19], following a gamma distribution whose mean and variance increase systematically with the mean delay [15]. This accounts for the variability of transmission delays through axons with heterogeneous properties (see Materials and Methods for the definition of the gamma distribution parameters). The mean inter-areal delay shown in the figures, hereafter termed 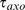, accounts for the latency between the generation of a spike in a presynaptic neuron from one network and the elicitation of a postsynaptic potential in the other network.

**Figure 3:**
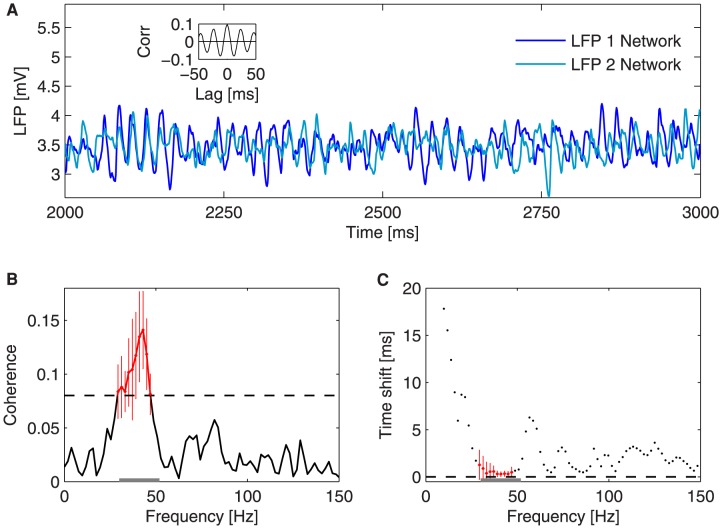
Collective oscillations of two coupled bidirectionally neural populations. The inter-areal axonal delay 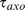 between the two neuronal pools is zero. (A) LFP time trace of the two populations in a 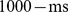 interval, for an external mean rate of 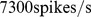. The inset shows the averaged time correlation of 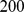 LFP pairs. (B) Phase coherence between the LFPs of the two networks for varying frequency. The measure is averaged over 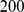 trials. The black dashed line represents the threshold (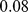) above which the phase coherence is considered significant (in red). (C) Time shift between the LFP oscillations of the networks for varying frequency. Red crosses show the time shifts corresponding to the frequencies at which the phase coherence is above threshold. The time shift is calculated as 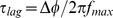, where 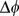 is the phase difference at the frequency 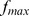 of maximum phase coherence. The gray bar delimits the gamma peak band (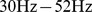). The measure is averaged over 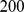 trials.

When coupled, the LFP power spectra of the two networks show the same gamma profile as in the absence of coupling, while the corresponding time series exhibit a substantial degree of correlation (Figure 3A inset). We next asked whether the broad spectrum of these neuronal oscillations allows for partial phase coherence to arise in specific frequency regions. Our phase coherence measure, described in the Materials and Methods section, quantifies between 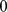 and 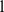 the reliability of the phase difference 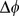 between pairs of oscillations, at a given frequency. Figure 3B shows the phase coherence between the LFPs of the two populations for instantaneous coupling (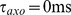). According to the regions of statistical significance observed experimentally [30], we considered phase coherence values above 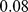, which mainly occurs within the gamma band around the peak of the LFP power spectrum (horizontal gray bar in Figure 3B). This threshold corresponds to around four times the average phase coherence of the uncoupled case (see black dashed line in Figure 3B).

We have also computed the time lag 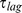 between the two signals (i.e. the time shift separating two equal phases of the coupled LFPs arising from each population) for all frequencies (Figure 3C), still in the case 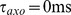. This time lag is only meaningful for significant phase coherence values that lead to a consistent 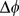 across trials (red crosses in Figure 3B). The figure shows that for frequencies at which the phase coherence is significant, the LFP gamma rhythms of the two populations oscillate in phase (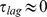), i.e. the two LFPs are synchronized at zero lag. The error bars in Figure 3B,C represent the standard deviation across trials of phase coherence and 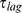 respectively, and are only shown for the region of significant phase coherence, since outside that region the phase distribution is very broad due to the variability across trials. Even within the significant region the standard deviation of 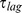 can be seen to decrease with increasing values of phase coherence, which confirms the inverse relation between phase coherence and the broadness of the phase distribution.

### Phase-coherence transitions for increasing coupling delay

**Figure 4:**
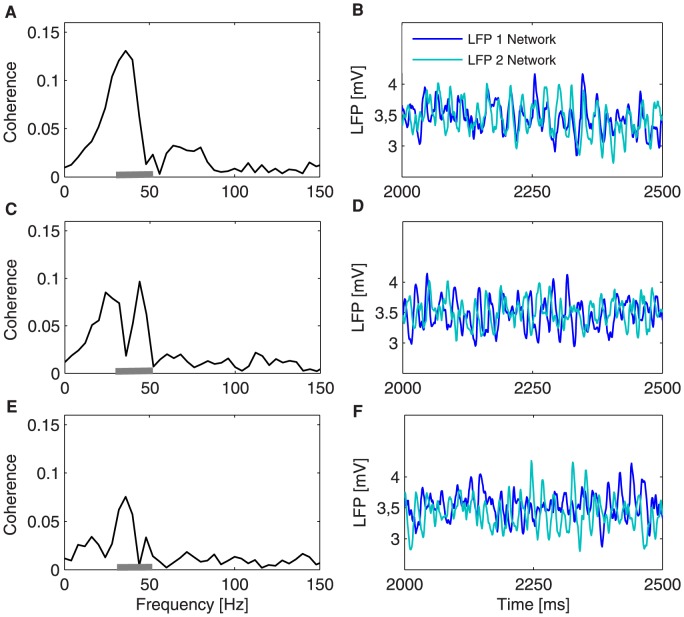
Phase coherence of two coupled bidirectionally neural populations for three different values of the inter-areal axonal delays 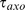. Phase coherence spectrum and corresponding representative time series for 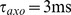 (A,B), 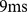 (C,D), and 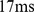 (E,F). The inter-areal delays follow a gamma distribution with a mean equal to corresponding inter-areal axonal delay 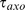. The gray bars on the x-axes of plots A, C, and E delimit the gamma peak band (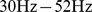). The phase coherence measure is averaged over 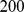 trials.

The fact that the two populations synchronize at zero lag when the coupling delay is zero is to be expected, and we now ask what happens in the presence of time delays. Figure 4 shows the phase coherence spectrum between the LFP oscillations for three different values of 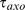. While phase coherence is again significant only around the gamma band (Figures 4A,C,E), the time traces look very different for small and large delays, with mostly in-phase dynamics for small delays (Figure 4B), whereas the populations are mostly in anti-phase for large delays (Figure 4F). For intermediate delays, interestingly, two coherence peaks appear (Figure 4C), and the corresponding time series exhibit both in-phase and anti-phase episodes (Figure 4D). These results indicate that in-phase dynamics seems to persist for non-zero coupling delays, eventually transitioning to an anti-phase regime with smaller, although still significant, phase coherence. Both types of dynamics seem to coexist for intermediate delays.

**Figure 5:**
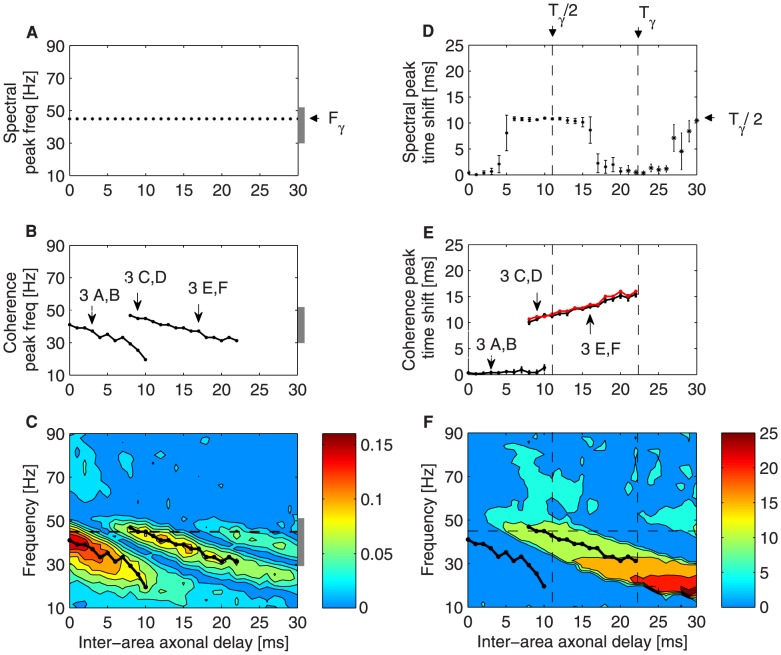
Phase coherence and time shift behavior in the case of bidirectional symmetric coupling for increasing inter-areal axonal delays 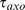. (A) Frequency 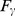
(black arrow) at which the power spectrum is maximum and extent of the gamma peak (gray bar) (results for only one population are shown, since they are the same for both populations). (B) Frequencies at which the phase coherence exhibits local maxima, 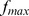. (C) Phase coherence, in color code, as a function of frequency (y-axis) and of the inter-areal axonal delay 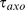 (x-axis). (D) Time shift 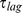 at the peak frequency 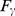 of the power spectrum. (E) Time shift 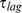 at 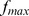, the frequencies labeled in (B). The red line corresponds to 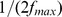. The labels in panels B and E correspond to panels of Figure 4. (F) Time shift 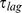, in color code, as a function of frequency (y-axis) and of the inter-areal axonal delay 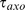 (x-axis). The solid black lines in panels C and F show 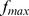 (as in panel B) and the dashed black line represents the power spectrum maximum within the gamma range shown in panel A. In plots A, B, and C the total extent of the gamma peak is displayed as a vertical gray bar. In plot D, the arrows point at the gamma period and half of it, 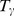 being 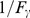. The measures are averaged over 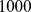 trials for each 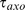.

In order to verify these conclusions, we have extended the analysis to a range of axonal delays, from 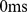 to 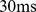, calculating the phase shift for the frequencies corresponding to both the peak of the power and the phase coherence spectra, termed 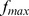. Figure 5A shows the value of the frequency at which the power spectrum is maximum, 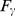, as a function of the coupling delay 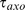. Note that varying 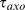 does not change the frequency peak of the LFP power spectrum, which remains around 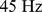 for all coupling delays. We have added a gray bar delimiting the maximum power spectrum range within the gamma band corresponding to the extent of this local peak, highlighting the fact that the LFP gamma rhythm expands over a range of frequencies between approximately 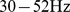.

On the other hand, 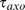 clearly affects the frequency 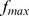 at which phase coherence is maximal, as shown by Figure 5B. In particular, 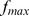 exhibits a discontinuous jump around a coupling delay 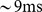, where two peaks of phase coherence coexist (consistent with the result shown in Figure 4C). The phase coherence values themselves are shown in color code in Figure 5C for different frequencies (vertical axis) and for varying 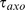 (horizontal axis). We have superimposed in that plot the line shown in panel A, which marks the maximum of the LFP power spectrum (black dashed line) within the gamma range, 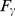, as well as the whole extent of the peak (vertical gray bar). The local peaks of phase coherence 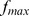 (black lines) corresponding to panel B are also superimposed to Figure 5C.

For 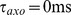 (as in Figure 3) the peak of phase coherence almost coincides with the peak of power spectrum. For increasing 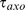, below 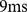, only the coherence peak at the lower frequency is significant, whereas between 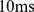 and 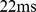 only the coherence peak at the faster frequency is above threshold. The transition between these two regimes involves a coexistence of the local coherence peaks. We also observe that in both branches the frequency at which phase coherence is maximum 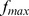 decreases with the axonal delay, becoming clearly smaller than the gamma frequency peak 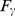 (dashed black line in Figure 5C). Making 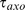 greater than 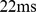, which approximately matches the period of the power spectrum peak 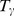 (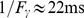), a new branch of phase coherence appears, thus leading again to coexistence of the two regimes. This emerging pattern is shown in Figure 5C for large inter-areal axonal delays and it is not marked in Figure 5B because the phase coherence is under the threshold. Hence, as 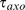 exceeds 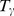, the scenario of relative phases is repeated but now with cycle skipping.

The phase coherence patterns shown in Figure 5C are affected by the inter-areal delay variability. If 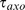 is fixed to a constant value, the region of coexistence between the in-phase and anti-phase coherence patterns increases, and for delays approaching the oscillation period 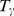 the new peak emerging at 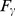 (detectable in Figure 5C and corresponding to in-phase dynamics in Figure 5E) becomes significant. This is shown in Supplementary Figure S1C, which displays the phase coherence for constant 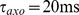 (blue line), in comparison with the case 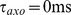 (black line) and the one with 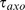 drawn from a gamma distribution with mean 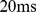 (red line).

According to Figure 5C, maximum values of phase coherence 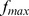 appear at different frequencies for each 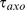. Significant values of phase coherence at a certain frequency can occur provided that there is a certain amount of spikes being simultaneously and reliably sent between the two networks. Since, by construction, the two neuronal pools are identical, the information flow can only be symmetrically transmitted for an in-phase, 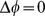, and/or an anti-phase, 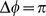, relationship between the two LFPs. Therefore, for any 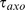 we can obtain the corresponding 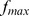 that satisfies 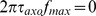 or 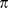. From this expression we can thus expect that larger 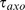 leads to smaller 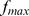 and that the anti-phase configuration is given by 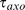 equal to half the period corresponding to 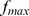, not to be mistaken with 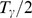, half the gamma period and equal to 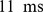.

To verify the aforementioned remarks we have next calculated the time shift 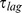 between the two coupled LFPs as 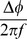. Figure 5D shows that, at the peak of the LFP power spectrum (here 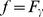), 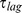 is zero for low (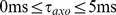) and large delays (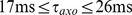). On the other hand, for intermediate (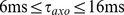) and large delays (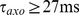) 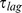 corresponds to half the period of the gamma rhythm (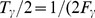) 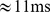). As mentioned before (see Figure 2), at frequency 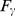 the MUA and the LFP in each population are frequency locked. Therefore, for any axonal delay, the presynaptic spikes arrive within the troughs of the postsynaptic LFP. We can interpret these sharp transitions from in-phase to anti-phase oscillations, appearing with a periodicity given by 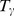, as the way by which the system keeps the symmetry for any 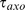.

Since the maximum of phase coherence 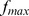 does not coincide with 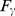, we have also obtained 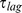 along the peaks of phase coherence. Figure 5E confirms that only two patterns arise: in-phase and anti-phase, which can simultaneously occur in the region between 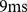 and 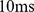. The lowest frequency branch corresponds to 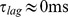, and thus to zero-lag synchronization. On the other hand, the highest frequency branch corresponds to a 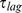 value that matches half the period of the corresponding frequency, i.e. 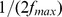 (labeled by a red line in the plot), and thus corresponds to anti-phase synchronization.

The full values of the time shift for all frequencies are shown in color code in Figure 5F. The region of zero-lag synchronization disappears as the delay increases, giving way to a region of anti-phase synchronization. Due to the oscillatory dynamics, for 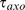 greater than 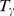, frequencies close to the gamma peak are again compatible with an in-phase pattern. However, it is important to note that phase coherence is strongly decreased as the cycle is repeated again (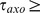 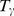), probably due to loss of temporal self-coherence of the oscillations themselves.

Thus, provided that the LFP-LFP phase coherence is significant, an effective coupling exists at which the two populations oscillate with a constant phase difference, which depends on the frequency and on the axonal delay. In particular, only two possible phase shifts are allowed, namely zero-lag (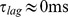) and an anti-phase (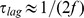) synchronization.

Figure 5C shows that the frequency at which maximum phase coherence occurs, 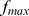, might not correspond to the predominant gamma rhythm at 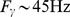, although it is close to it and within the extent of the gamma peak (gray vertical bar). Thus, phase coherence is bounded by the region in which spikes are still phase locked to the LFP (Figure 2). The separation between 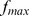 and 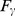 is clear when 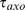 varies between 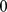 and 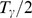. Phase coherence is achieved at slower rhythms that still reliably carry the action potentials. Hence, the spikes elicited by each population systematically reach the other one at its excitability windows. Moreover, lower 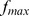 implies larger excitability windows and allows the networks to be synchronized in phase. For larger 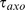, corresponding slower frequencies lying outside the gamma peak do not efficiently transmit spikes, due to the bounded region in which MUA is locked to the LFP. Therefore, at large 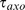 the system moves towards an anti-phase configuration, where the time lag matches and compensates for the inter-areal axonal delay.

### From phase coherence to communication

The LFP oscillations studied so far are complex rhythms that convey a wide range of frequencies with a predominant component in the gamma range. We have seen before that the axonal delay 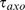 determines the relative dynamics of the coupled neuronal pools, which fall in either an in-phase or an anti-phase pattern. The phase relationship set by the two LFP signals is proposed to regulate the effectiveness of communication [2]. In other words, a stable phase difference 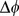 determines the response of a neuronal population to inputs perturbing directly another area. Therefore, depending on the phase difference 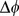 between two coherent LFPs, the response of the unperturbed population will replicate to a certain extent the response of the other population to the perturbation. We next study how, in the two different synchronization scenarios described above, inter-areal axonal delays affect information transmission during temporal windows, in which the phase difference and the frequency cannot be independent of each other. Note here the difference between phase coherence and effective communication. Rigorously speaking, communication occurs whenever spikes from one population arrive to the other one, and this is guaranteed provided that there is some coupling across networks. In contrast, effective communication refers to a more specific situation in which information about the stimulus is being carried by the coupled populations.

We can obtain a good proxy for communication by quantifying the response of a neuronal population (the receiver) to a perturbation that affects indirectly its dynamics via a second population coupled to it (the emitter), and which receives directly the perturbation. The success in communication can be observed in the transient amplification of the neuronal oscillations of the receiver [31]. The perturbation simulates different stimulus features, and consists of increases in the mean firing rate of the background synaptic activity impinging on a subpopulation of the emitter. We have examined, at different inter-areal axonal delays 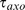, how well the LFP and MUA power spectra of the receiver convey information about the external stimulus being applied to the emitter.

Since the connectivity within and between the two neuronal networks exhibits a certain degree of clustering, exciting a subpopulation of adjacent excitatory neurons from an area in the emitter population triggers a response in a well-defined subpopulation of neighboring neurons in the receiving population. We have chosen a set of different input intensities, 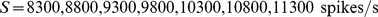, affecting 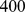 long-range excitatory neurons from the emitter population during a 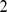-second time window. As a consequence of this perturbation, the amplitude of the LFP power spectrum increases with the strength of the perturbation (Supplementary Figures S2A,B, S3A,B and S4A,B), without altering the position of the gamma frequency peak (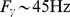), consistent with the results were reported in [20].

**Figure 6:**
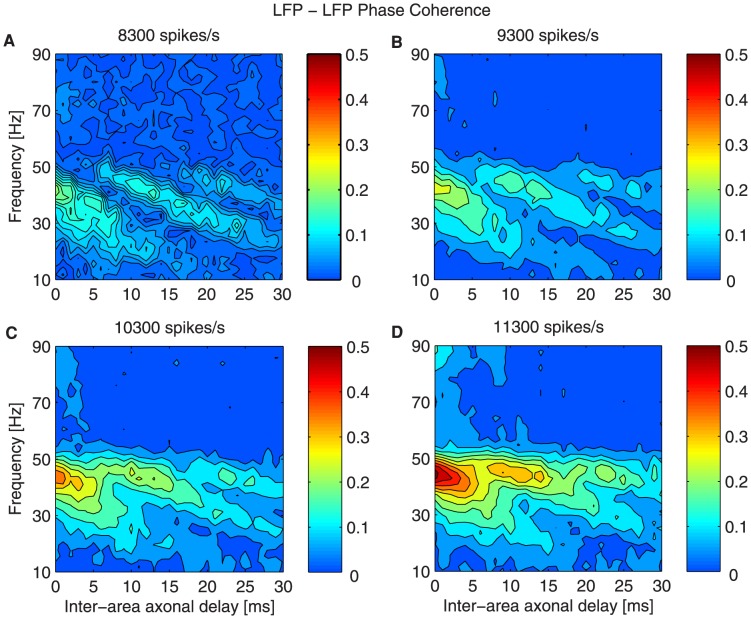
Phase coherence in the case of bidirectional asymmetric coupling for increasing extra inputs. Phase coherence between LFPs of the two networks, in color code, as a function of frequency (y-axis) and of the inter-areal axonal delay 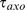 (x-axis) for different stimuli: (A) 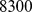, (B) 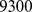, (C) 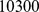, (D) 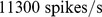. The measures are averaged over 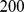 trials for each 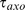 and stimulus.

**Figure 7:**
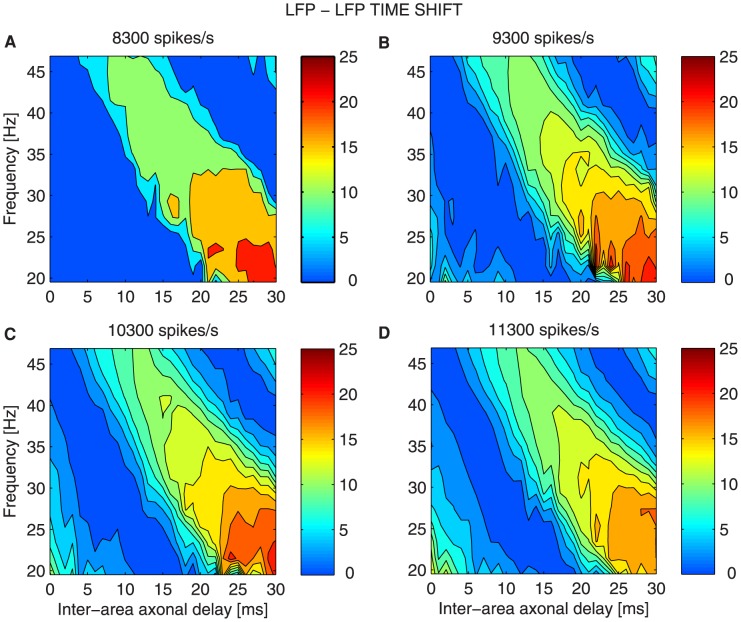
Time shift in the case of bidirectional asymmetric coupling for increasing extra inputs. Effective time shift in milliseconds between LFPs of the two networks, in color code, as a function of frequency (y-axis) and of the inter-areal axonal delay 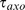 (x-axis) for different stimuli: (A) 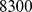, (B) 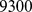, (C) 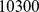, (D) 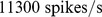. The measures are averaged over 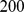 trials for each 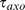 and stimulus.

Perturbing one of the populations breaks the symmetry of the system, since now the firing activity of the emitter is enhanced with respect to the receiver. As shown by the maps of phase coherence plotted in Figure 6, an increase of the external firing rate boosts phase coherence between the two LFPs. Moreover, the two frequency bands where phase coherence is significant merge into a single region at larger values of 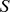 concentrating closer to the gamma frequency peak 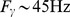. The corresponding 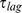 values are shown in Figure 7 (note the different ranges of the axes, which now concentrate on the significant values of phase coherence to better observe the transition to the out-of-phase regime).

**Figure 8:**
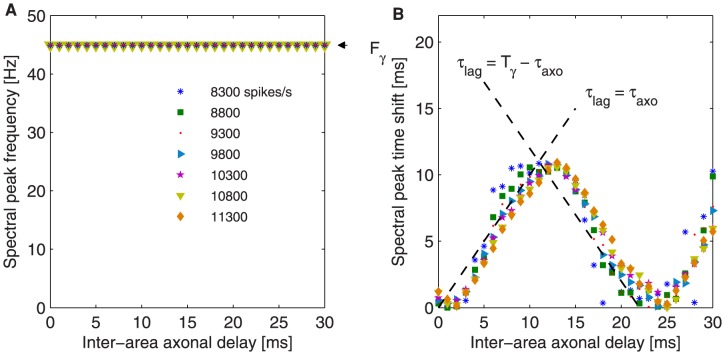
Time shift behavior at the peak of power spectrum for increasing inter-areal axonal delays for different extra inputs. Effect of the external input perturbation on the coupled neuronal populations for increasing stimulus strengths 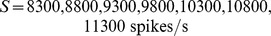. (A) Frequency of the power spectrum peak. (B) Time shift corresponding to spectral peak frequency. The dashed lines show the ideal cases for which 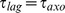 and its anti-phase equivalent.

At the gamma frequency peak 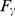 the system undergoes a transition from in-phase to anti-phase dynamics as 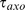 increases. Small 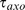 lead to time shifts 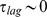 of the emitter's LFP relative to the receiver's LFP (Figure 7A-B) and thus, the two signals oscillate approximately in phase. However, the route to the anti-phase configuration changes as 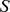 is strengthened. In particular higher 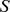 trigger smoother transitions and the anti-phase regime becomes narrower. Figure 8B shows 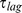 values tracked at the gamma frequency peak 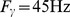. Here, larger 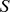 leads to a leader-laggard configuration in which the emitter LFP precedes the receiver LFP by a time lag that equals the axonal delay (see dashed black lines). Supplementary Figures S2C,D, S3C,D and S4C,D show the phase coherence and time shift for 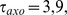 and 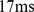 (the same delays as Figure 4) for the whole range of frequencies.

The dependence of the phase coherence on 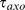 for different 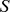 values is shown in Figure 6A–D, corresponding to a shift from a symmetric to an effectively asymmetric coupling. As the extra perturbation is applied only to one of the populations, the effective coupling approaches an unidirectional connectivity, although the structural links are not changed. This can be further explained by carrying on the same analysis in a structural unidirectional scenario, in which only one population projects onto neurons from the other network. Supplementary Figure S5A shows that increasing the delay 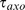 of the unidirectional transmission, the networks keep the phase difference constant at approximately the same frequency close to the power spectrum peak frequency. This represents a leader-laggard configuration and is similar to what happens in Figure 6D, where an over-excited subpopulation is driving the coupling between the two networks, still bidirectional but strongly asymmetric. The decrease of phase coherence with axonal delay is due to the variability in delay times: fixing 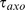 to a constant value of 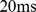 leads to maximal phase coherence values comparable to the case of no delay (Supplementary Figure S1A). Figure S5B shows that for increasing inter-areal axonal delays 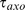, the time shift between the two synchronized networks (at frequencies corresponding to the significant phase coherence of Figure S5A) increases as long as 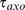 is smaller than half the period of LFP oscillation (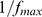) and then approaches zero, thus leading again to a transition from in-phase to anti-phase synchronization at frequencies close to that of the power spectrum peak 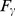.

Phase coherence can influence the transmission of information between neuronal populations. As mentioned in the Introduction, the CTC hypothesis [2] suggests that phase relations between coupled areas modulate the response of a receiver area to presynaptic input coming from an emitter area. In order to maximize this response, the axonal delay 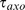, the frequency 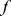 of the oscillations and the phase difference 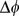 should verify 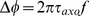. When this relationship holds, spikes fired in the emitting population at a specific phase of the signal (for instance at the troughs of the LFP, which correspond to the maxima of excitability) arrive at the receiving area at the same phase (and thus at the same excitability maximum), triggering a maximal response in the receiving area. On the contrary, if 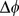 does not fulfill the relationship given above (or if it randomly varies), effective communication will not be achieved [31]. This condition is relevant at the frequencies at which the MUA and the LFP are phase locked (Figure 2). Otherwise, the troughs of the LFP do not correspond to intervals of maximum firing within the same population, and the peaks of MUA do not occur reliably with the same periodicity as the LFP.

In order to quantify the efficiency of communication, we have computed the mutual information 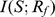 (defined in the Material and Methods section) between the power spectrum 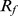 at a frequency 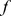 of both the LFP and MUA of the receiver and the set of stimuli 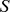 applied to the emitter. This definition of information does not require any assumption about the stimulus features being encoded by the neural signals [32], [33]. 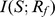 quantifies the reduction of the uncertainty in predicting the applied stimulus given a single observation of the triggered response, and is measured in units of bits (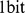 means a reduction of uncertainty of a factor of two). Several experiments have been done with this tool to characterize information transmission in the primary visual cortex of macaques in response to a naturalistic stimulus [33]. Several other studies have been performed using the LFP power spectrum as a measure of mutual information, showing the usefulness of this approach both experimentally and computationally [20]. The advantages of this approach are described in detail in [34], [35].

**Figure 9:**
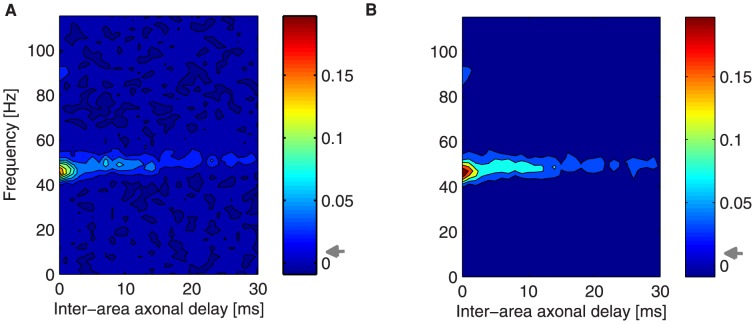
Mutual information carried by LFP and MUA power spectrum of the receiver. Mutual information between the set of stimuli 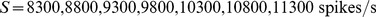 and the neural response given by the LFP (A) and MUA (B) power spectra for increasing coupling delays 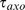. The gray arrow in the color scale refers to significance threshold (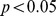, bootstrap test). The measures are averaged over 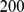 trials for each 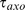 and stimulus.

To compute 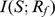, we have run extensive simulations to properly estimate the conditional probabilities used in mutual information measures. The techniques adopted in order to reduce the bias error of the estimation of conditional probability due to the finite number of samples are explained in the Material and Methods section. Figure 9 shows that the mutual information is non-negligible only within the gamma range (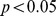; bootstrap test), in a narrow region around the peak of the power spectrum 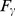. This is consistent with the fact that the emitter encodes the different stimulus strengths in the gamma band, i.e. other regions of the LFP power spectrum are not affected (Figure S2–S4A). Therefore, information transmission occurs within the gamma peak (the mutual information spectrum of the two networks, computed from the LFP, for 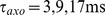 is plotted in Supplementary Figures S2E–S4E). Moreover, functional interactions between coupled neuronal populations can be maximized if their phase difference is close to zero, i.e. for short axonal delays.

While 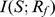 is lower when computed for the LFP power spectrum (Figure 9A) than for the MUA power spectrum (Figure 9B), it decreases monotonically in both cases for increasing axonal delays. This behavior contrasts with the one shown in Figure 5C, in which the maximum phase coherence in the absence of stimulus occurs at varying frequencies 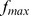 for different 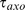. Moreover, 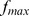 lies outside the significant mutual information spectrum. However, at large enough 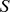 the phase coherence pattern (Figure 6D) closely resembles the mutual information dependency with 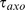 (Figure 9), since here 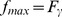.

Mutual information encoded in the power spectrum is bounded to the frequencies at which spikes are maximally frequency locked (Figure 2). Although this measure does not take into account the phase difference between the two LFP signals, their dynamics clearly rely on their relative time lag. Therefore, significant phase coherence is needed in order to reliably connect in time the excitability time windows of both networks, but it is not sufficient to achieve a maximal response of the receiver. In order to meet this second requirement, the frequency at which phase coherence is obtained needs to carry a precise timing of the action potentials, otherwise the presynaptic current will not elicit a postsynaptic response. Even the emitter population can only encode the stimulus strength as variations in the amplitude of the gamma frequency peak, since it is at 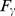 that changes in the LFP represent synchronized alterations in the MUA.

A symmetric coupling scenario allows for two emerging stable regimes, in-phase 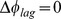 and anti-phase 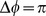, while in an asymmetric regime the most excitable network leads the dynamics (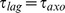). Therefore, in the presence of axonal delays, the latter case is not compatible with the in-phase/anti-phase condition. The symmetry breaking allows for 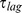 to follow 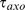, increasing phase coherence at the gamma rhythm and thus the receiver's response. In summary, efficient communication needs a sufficient locking between the spikes being transmitted and the LFP, the carrier of this information. This is maximized at the gamma frequency peak 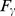, here 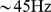, at which changes in the power spectrum due to external stimuli become particularly evident. The coupling axonal delay 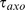 modulates the level of phase coherence within all the gamma range, and strong driving of one of the populations precisely favors the 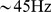 frequency channel. As observed above, the variability of axonal delay affects the regions where the phase coherence maximum is significant. Supplementary Figures S6A,B show the LFP and MUA mutual information in the unidirectional case. As in the case of bidirectional coupling, the flow of information occurs at 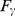, where the MUA and LFP are frequency locked and the emitter encodes the stimulus strength. Specially, mutual information is higher at small 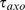, when the networks are synchronized in phase. In the unidirectional configuration the mutual information shows a strong dependence on 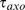, as in the case of phase coherence discussed above. This is due again to the variability of axonal delays. For a fixed time delay, the mutual information in the unidirectional coupling case does not show a substantial decrease for increasing 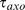 (Supplementary Figure S1B). The bidirectional configuration also exhibits a less significant decrease of the mutual information maximum for constant increasing 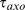 (Supplementary Figure S1D). This is consistent with the phase coherence peak corresponding to in-phase dynamics that persists for increasing constant delay (Supplementary Figure S1C).

Our results show that phase coherence cannot be taken as a precursor of information transmission. Phase coherence can be achieved in a broad range of frequencies around the gamma peak 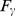 (Figure 6). Therefore, the spikes impinging on each network are able to bound the two populations in a constant phase relationship, constrained by the symmetry of the effective coupling. However, in order to communicate, presynaptic spikes must trigger a postsynaptic response. This requires that the presynaptic action potentials are synchronized in time to facilitate the integration of the synaptic currents. Hence, changes in the LFP and MUA amplitude occur precisely at 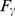 and mutual information does the same (Figure 9). Stimulus that are able to modify the response of a population within a wider frequency range (i.e. not frequency specific) could possibly alter the situation here described.

## Discussion

Here we have examined how heterogeneous inter-areal synaptic delay influences the coupling between the collective dynamics of two neuronal populations. To that end, we first used population measures such as the local field potential and the multi-unit activity, by analogy with experimental studies, to capture the collective oscillatory dynamics of individual neuronal populations. In the presence of excitatory coupling, the LFP and MUA activities of two identical delayed neuronal networks oscillate in the gamma range, with a significant broad peak between 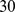 and 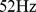, which does not depend on the axonal delay 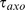. The emergence of this gamma peak in the isolated populations is due to the recurrence between excitatory and inhibitory synaptic activity, as revealed by the phase locking between the LFP and MUA signals (Figure 2). In contrast with the power spectrum, phase coherence is strongly affected by the axonal delays between the populations. We have seen that in-phase and anti-phase patterns occur at various frequencies for different ranges of 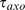, with high values of phase coherence occurring at frequencies below the gamma frequency peak 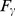 (Figure 5).

The phase coherence pattern shown in Figure 5C corresponds to a pure symmetrical connectivity, in which both the structural and functional coupling are equal in both directions (in contrast with the unidirectional case of Figure S5). The reciprocity between the feedback and feedforward pathways across cortical areas is not an unrealistic assumption [36], although the specificity of such synapses might differ in each direction in order to account for the different effects of the top-down and bottom-up projections. Here we show that increasing axonal delays 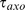 lead to either an in-phase or anti-phase synchronization with a vanishing maximal phase coherence at frequencies 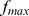 below 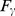 although lying within the gamma peak. Hence, in *basal conditions*, there is always a certain reliable phase relationship, provided 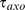 is small, relative to the period 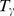.

The interesting point raised by the communication through coherence hypothesis [2], is whether phase coherence can forecast efficient communication between two populations in the presence of a stimulus. According to the modulatory role of the top-down pathway, attention can determine which visual cues we are aware of [37], [38]. In principle two situations can arise: either a stimulus catches our attention (such as an unexpected noise or object) or we are being attentive to an expected stimulus (such as waiting the traffic light to turn green). In the first situation the communication outline between a primary cortical area and the associative areas is driven by the stimulus, while in the second case it is due to the internal cognitive state. The firing activity in visual areas has been shown to significantly increase even in the attentive state prior to the stimulus presentation [39]. Hence our results, in which we progressively increase the firing rate impinging on one population, could be viewed as arising from the alteration of the underlying attentional state.

The experimental study of [38] shows that a neuronal cell assembly in V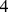 is phase coherent with an area in V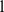 that responds to a selected stimulus, while it is not with a V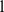 area that is not driven by the input. Here we have not modeled a competitive scenario between two networks. Instead we have focused on the mechanisms by which two neural pools can modulate their communication when they are simultaneously oscillating in the gamma band. We have quantified the efficiency of communication between the two neuronal networks as the ability of a population to encode information of an input which perturbs directly another coupled population. Mutual information measures between either the LFP or MUA power spectrum and the set of applied stimuli 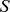 show that significant values concentrate around the gamma frequency peak (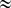 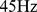). Mutual information decreases for long inter-areal axonal delays, and is slightly lower when the neural response is characterized by the LFP power spectrum than by the MUA power spectrum.

Despite the fact that the LFP reflects the afferent and local synaptic currents within a given neuronal network, and that the MUA only captures the action potentials within this network, these two signals are closely related. As mentioned above, the gamma LFP rhythm reflects the dynamics of the excitatory balance. Increases in inhibition silence the spiking activity and therefore the MUA signal, although the GABAergic current is enhanced. Decreases in inhibition boost the spiking activity and therefore the MUA signal, although the GABAergic current is reduced. The peak at 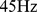 in the LFP-MUA phase coherence (Figure 2) reveals this phase locking between the two signals.

The arrival of each set of presynaptic spikes perturbs the postsynaptic LFP and might or might not elicit a postsynaptic suprathreshold response (captured by the postsynaptic MUA) depending on the mean distance to the excitatory threshold of the populations. Bursts of activity occur at each pool with a periodicity that fluctuates within the gamma band and are locked to the troughs of the LFP at this frequency. According to the CTC hypothesis, maximum communication requires that spikes from each population reach the peaks of excitability of the target area simultaneously in both coupling directions. Thus, efficient communication is restricted to the gamma peak, as revealed by the mutual information (Figure 9) and preferentially at relatively small 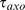. This condition is only met for in-phase or anti-phase synchronization of the gamma rhythm: small axonal delays 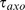 are able to tie two LFP troughs only at zero-lag synchronization, while larger 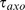 require anti-phase synchronization. In principle, as 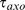 increases zero-lag synchronization could again mediate communication by skipping one cycle. However, due to loss of phase consistence, mutual information decays with increasing 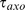.

Here we show that phase coherence emerges spontaneously due to the excitatory coupling between areas without the need of further constrains (Figure 5C). Higher stimulation of a particular population (the emitter), which enhances the LFP power spectrum amplitude of the gamma peak, increases the range of phase coherence to larger axonal delays (Figure 6D). The delay determines the phase shift between the two signals, with the emitter leading the oscillations. According to [38], phase coherence is revealing communication in the sense of spike propagation, which in our case extends to frequencies within the gamma peak. However, *efficient communication* in the sense of the information encoding in the postsynaptic response, is restricted to a narrower band (Figure 9) that maximizes spike synchronization. Adopting a spectrum of delays with increasing variability for increasing values of 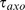, instead of an (unrealistic) constant delay, affects quantitatively the results of phase coherence and mutual information but does not produce any strong qualitative change in the findings of the paper. However the effect of variability cannot be ignored, given the dispersion of axonal delays observed in experimental studies [19].

**Figure 10:**
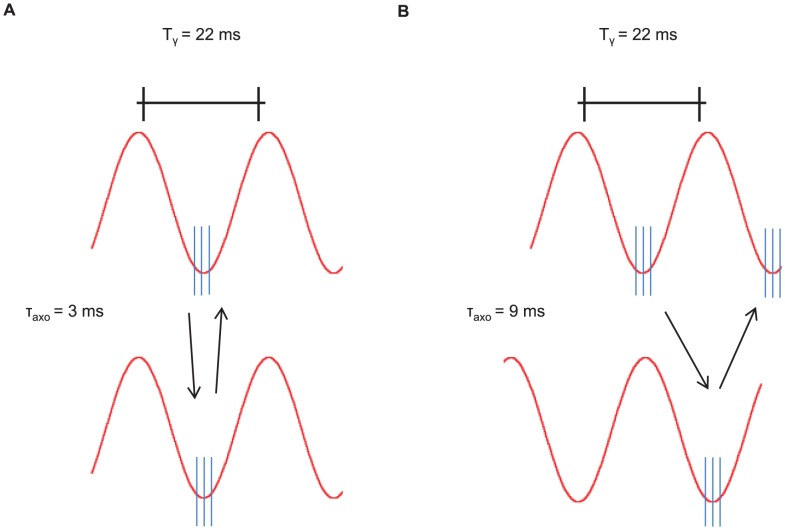
Carriers of information and signals. Diagram of two oscillatory LFPs filtered around the power spectrum peak (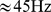), with a short spike train locked at their troughs for different 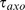: (A) 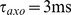, representing zero-lag synchronization and (B) 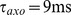, representing anti-phase synchronization.

Figure 10 shows a schematic diagram of the two oscillatory LFPs filtered around the gamma frequency peak (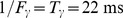) with the bursts of spikes locked at their troughs in agreement with Figure 2. For a delayed coupling, zero-lag synchronization does not lead to a symmetric configuration demanding that the two oscillations are reciprocally influenced at the same phase. Therefore the system rapidly shifts toward an anti-phase synchronization at which 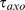 roughly equals half of the period of the LFP (Figure 10B). Importantly, when the symmetry of the system is broken (for instance by perturbing just one of the populations), the possible stable solutions are no longer the in-phase or the anti-phase regime. In this case, phase coherence can be achieved through a leader-laggard configuration in which the time lag equals the inter-axonal delay. Without the symmetry constraint, this situation is achieved at the gamma frequency peak, for which the spikes of each population are preferentially locked to the LFP and changes in their power spectrum are maximized.

In conclusion, we have studied two neuronal populations coupled synaptically with non-negligible delays. Our modeling results show that the populations organize their joint collective dynamics in patterns of in-phase or anti-phase synchronization, depending on the delay. Unidirectional couplings, either structural or functional, lead to a leader-laggard configuration with an out-of-phase synchronization determined by the axonal delay. Our study shows the dichotomy between phase coherence and communication. Whereas phase coherence arises due to LFP phase perturbations through the propagated spikes, communication is caused here by an increase in the firing response. The first occurs at different frequencies for every 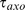 in order to conserve the functional connectivity. The second requires the spikes to be tightly locked to the LFP and at a faster frequency 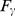 to enable spike integration, and hence a signal response that can be synaptically propagated.

## Materials and Methods

### Computational model

We consider two populations of 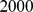 neurons, 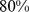 of which are excitatory while the remaining 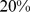 are inhibitory [40]. Each neuron connects on average with 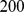 other cells through only chemical synapses. The structural connectivity is built according with the Watts-Strogatz small-world algorithm [41]. The rewiring probability is set to 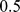, so that the connectivity shows a certain degree of clustering, which favors the connections between neighboring neurons. Coupling between the two networks is mediated by 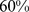 of the neurons of one population making random long-range excitatory projections with 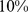 of the neurons belonging to the other population. Here we assume that the connectivity within a network is 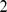-fold the connectivity across networks, neglecting heterogeneity across neurons. Moreover, in order to obtain a certain amount of phase coherence between the two networks, we consider that the majority of excitatory neurons project onto the other network. A stronger (weaker) coupling will lead to unrealistically higher (lower) phase coherence values [30]. We introduced a synaptic transmission delay within and among the networks, taken from a gamma distribution, assuming that internal delays (taken from a gamma distribution whose scale and shape parameters are fixed to 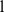) are smaller than the inter-area delays. The axonal delays, termed 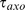 in the paper, stand for the time between the generation of a spike in a presynaptic neuron from one network and the elicitation of a postsynaptic potential in the other network. These delays are taken from a gamma distribution whose mean and variance increase with increasing 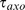. We choose the scale parameter of the distribution equal to unity, so that the shape parameter equals 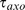. In this way the coefficient of variation (CV) decreases for increasing mean as 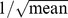. In our analysis we systematically vary 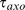 between 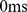 and 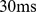.

Each neuron is dynamically described by the Hodgkin and Huxley (HH) model. The dynamics of the membrane voltage is given by: 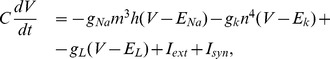
(1)where 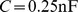 (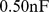) is the membrane capacitance for inhibitory (excitatory) neurons, the constants 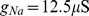, 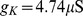, and 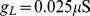 are the maximal conductances of the sodium, potassium, and leakage channels, respectively, and 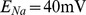, 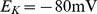, and 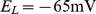 stand for the corresponding reversal potentials. According to the HH formulation, the voltage-gated ion channels are described by the following set of differential equations



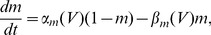
(2)

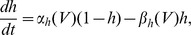


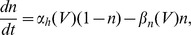
where the gating variables 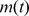, 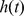 and 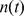 represent the activation and inactivation of the sodium channels and the activation of the potassium channels, respectively. The voltage-dependent transition rates are



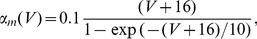
(3)

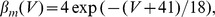


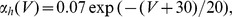


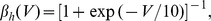


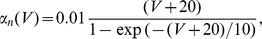


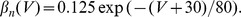



Given that 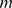 activates rapidly, we replace it by its steady-state value 
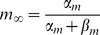
.

In Equation (1) 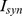 is the synaptic current coming from the neighboring neurons impinging on a neuronal cell: 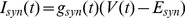
(4)where 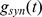 is the synaptic conductance and 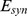 is the reversal potential of the synapse. For positive values of 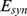 the synapse is depolarizing or excitatory (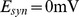 for glutamate receptors), otherwise it is hyperpolarizing or inhibitory (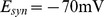 for GABA receptors). In the equation (4) the synaptic conductance is described by:
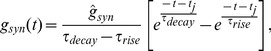
(5)where 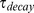 and 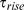 are the decay and rise synaptic time, respectively, and 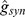 is tuned in order to obtain a balance between excitation and inhibition. The constant 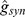 is set to maintain the postsynaptic potential (PSP) amplitudes within physiological ranges. All parameters values can be found in [26], [42].

In equation (1) 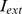 represents an heterogenous Poisson train of excitatory presynaptic potentials with a mean event rate that varies following an Ornstein-Uhlenbeck process (see Supplementary Figure S7). This incoming external current mimics the direct input from any other area external to the network considered here. The instantaneous rate, 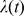, of the external excitatory train of spikes is generated according to an Ornstein-Uhlenbeck process, as considered in [20]: 
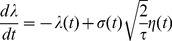
(6)where 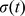 is the standard deviation of the noisy process and is set to 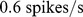. 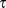 is set to 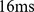, leading to a power spectrum for the 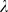 time series that is flat up to a cut-off frequency 
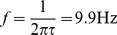
. 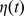 is a Gaussian white noise.

The model has been integrated using the Heun algorithm [43], with a time step of 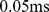. All simulations represent 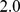 seconds of activity. The connectivity, initial conditions and noise realization were varied from trial to trial.

### LFP and MUA

We quantified the activity of the network in two different ways. We calculated the multi-unit activity (MUA) as the total number of spikes per unit time in the population, and the local field potential (LFP) as the sum of the absolute values of the excitatory and inhibitory synaptic currents acting upon the excitatory neurons, averaged over this population [20]: 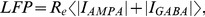
(7)where 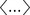 denotes the average over all excitatory neurons. The term 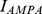 accounts for both the external excitatory heterogeneous Poisson spike train and the recurrent excitatory synaptic current due to the network, while 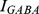 corresponds to the recurrent inhibitory synaptic current. 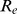 represents the resistance of a typical electrode used for extracellular measurements, here chosen to be 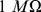.

### Spectral analysis

We computed the power spectral density of LFPs and MUAs using the Welch method: the signal is split up into 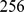 point segments with 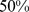 overlap. The overlapping segments are windowed with a Hamming window. The modified periodogram is calculated by computing the discrete Fourier Transform, and then computing the square magnitude of the result. The modified periodograms are then averaged to obtain the PSD estimate, which reduces the variance of the individual power measurements. The code has been implemented in MATLAB. Spectral quantities are averaged over 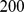 trials and phase coherence over 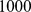 trials.

### Phase coherence

Phase coherence is calculated as in [30]: 
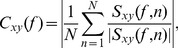
(8)where 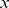 and 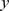 denote the two signals, and 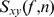 is the cross-spectrum between them. Since in each trial the cross spectral density is normalized by its amplitude, each term of the sum is a unit-length vector representation of the phase relation 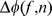. In other words, 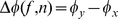 is the phase lag between the two signals at frequency 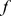 in the data segment 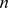. Hence 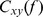 quantifies how broad is the distribution of 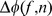 within the 2*π*-cycle. Averaging 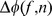 across all 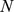 data segments provides a mean angle 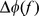. In our work 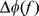 is converted into a time shift, termed 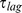 in the paper, dividing by the corresponding frequency 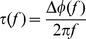. This quantity measures the time separation between an LFP maximum in one population and the following maximum belonging to the other population.

### Mutual information

An important mathematical tool to quantify information transmission in noisy systems is provided by information theory. We calculate the Mutual Information 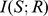 between the stimulus 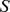 and the response 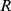 as follows. The broadband LFP signal reproduces the variations in neural population activity over a wide range of time scales [44]. Thus LFPs signals are useful to qualitatively characterize mechanisms of information processing, because it is possible through them to verify if there are priviliged time scales for information processing. We can think that information is spread over all frequencies, or that each frequency contributes separately to the information representation. Given that we are interested in how the collective dynamics of the population carries information, we quantify the neural response 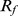 as the power of either the LFP or the MUA at frequency 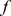, and we consider as stimuli different external firing rates impinging on one of the two populations. We compute the information between the stimulus 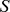 and the response 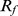 as: 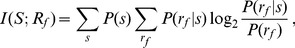
(9)where 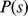 is the probability of presenting stimulus 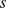 (equal to the inverse of the total number of different external firing rates, namely of stimuli), 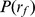 is the probability of observing power 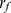 across all trials in response to any stimulus, and 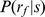 is the probability of observing power 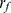 at frequency 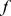 in response to a single stimulus 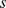. 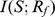 quantifies the reduction of uncertainty about the stimulus that can be gained from observing a single-trial neural response, and we measured it in units of bits (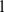 bit means a reduction of uncertainty of a factor of two) [35]. This measure allows us to evaluate how well the power 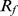 of either the LFP or MUA encodes the stimulus at a certain frequency 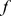.

To facilitate the sampling of response probabilities, the space of power values at each frequency was binned into 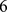 equipopulated bins [33]. We used seven different firing rates of the external Poisson-distributed input, for a time 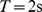. An important issue to be solved regarding the calculation of the theoretical mutual information is that it requires knowledge of the full stimulus-response probability distributions, and obviously these probabilities are calculated from a finite number of stimulus-response trials. This leads to the so-called limited sampling bias, which corresponds to a systematic error in the estimate of information. We used the method described in [45] to estimate the bias of the information quantity and then we checked for the residual bias by applying a *bootstrap procedure* in which mutual information is calculated when the stimuli and responses are paired at random. If the information quantity is not zero (it should be in the case of non finite samples), this is an indication of the bias and the bootstrap estimate of this error should be removed from the mutual information. After applying these procedures, the information quantity estimation could be defined as significant. Several toolboxes provide different bias-correction techniques, which allow accurate estimates of information theoretic quantities from realistically collectable amounts of data [46], [47]. In order to accomplish those tasks, we used the Information Breakdown Toolbox (ibTB), a MATLAB toolbox implementing several information estimates and bias corrections. It does this via a novel algorithm to minimize the number of operations required during the direct entropy estimation, which results in extremely high speed of computation. It contains a number of algorithms which have been thoroughly tested and exemplified not only on spike train data but also on data from analogue brain signals such as LFPs and EEGs [47].

## Supporting Information

Figure S1
**Phase coherence for constant inter-areal delay.** (a) Phase coherence between the two LFP oscillations in the unidirectional coupling configuration when 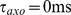 (black line), 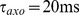 (blue line) and 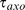 is taken from a gamma distribution of mean 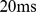 (red line). (b) Mutual information between the set of stimuli 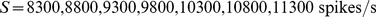 and the neural response given by the LFP in the same unidirectional coupling configuration. (c) Phase coherence and (d) mutual information in the bidirectional coupling configuration. Phase coherence measures are averaged over 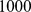 trials. Mutual Information measures are averaged over 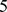 sets of 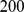 trials for each stimulus.(EPS)

Figure S2
**Effect of external stimulation for small coupling delay.** (a) LFP power spectrum of the directly stimulated population for different external rates (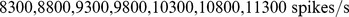). (b) LFP power spectrum of the second population. (c) Phase coherence between the two LFPs for different external rates. (d) Effective time delay between the two pairs of LFP oscillations at frequencies where the phase coherence is significant for different external rates. (e) Mutual information between the LFPs of the two populations. Red dashed line corresponds to significance threshold (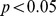; bootstrap test) for information. The mean inter-delay between the pools is 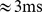. The measures are averaged over 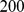 trials.(EPS)

Figure S3
**Effect of external stimulation for intermediate coupling delay.** The meaning of the plots is the same as in Suppl. Fig. S2. The mean inter-delay between the pools is here 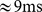.(EPS)

Figure S4
**Effect of external stimulation for large coupling delay.** The meaning of the plots is the same as in Suppl. Fig. S2. The mean inter-delay between the pools is here 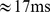.(EPS)

Figure S5
**Phase coherence and time shift in the case of unidirectional coupling.** (a) Phase coherence, in color code, as a function of frequency (y-axis) and of the inter-areal axonal delay 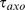 (x-axis) in the case of unidirectional coupling from the emitter to the receiver. (b) Time shift 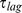, in color code, as a function of frequency (y-axis) and of the inter-areal axonal delay 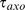 (x-axis) in the case of unidirectional coupling from the emitter to the receiver. The measures are averaged over 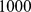 trials consistently with the symmetric coupling.(EPS)

Figure S6
**Mutual information in the case of unidirectional coupling.** Mutual information between the set of stimuli 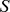 and the LFP (A) and MUA (B) power spectra for increasing coupling delays 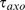 when the coupling is unidirectional from the emitter to the receiver. Note the different colorbar scales in the two cases. The gray arrow in the color scale refers to significance threshold (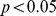, bootstrap test). The measures are averaged over 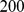 trials for each 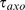 and stimulus.(EPS)

Figure S7 **External population input.** Time-varying rate of Poissonian spike trains representing the external inputs to a neuron in the network (black). The mean firing rate is shown in blue. The noise is modeled as an Ornstein-Uhlenbeck process.
(EPS)
